# Identification of the potential mechanism of *Radix pueraria* in colon cancer based on network pharmacology

**DOI:** 10.1038/s41598-022-07815-y

**Published:** 2022-03-08

**Authors:** Yi Li, Chunli Zhang, Xiaohan Ma, Liuqing Yang, Huijun Ren

**Affiliations:** 1 Department of Clinical Laboratory, The First Affiliated Hospital of Zhengzhou University, No. 1 Jianshe Road, Zhengzhou, 450052 People’s Republic of China; 2 Department of General Surgery, The People’s Hospital of Zhengzhou, Henan, China; 3 The Third Affiliated Hospital of Zhengzhou University, Henan, China; 4 Fuwai Central China Cardiovascular Hospital, Henan, China

**Keywords:** Cancer, Computational biology and bioinformatics

## Abstract

*Radix Puerariae* (RP), a dry root of *Pueraria lobata* (Willd.) Ohwi, is used to treat a variety of diseases, including cancer. Several in vitro and in vivo studies have demonstrated the efficacy of RP in the treatment of colon cancer (CC). However, the biological mechanism of RP in the treatment of colon cancer remains unclear. In this study, the active component of RP and its potential molecular mechanism against CC were studied by network pharmacology and enrichment analysis. The methods adopted included screening active ingredients of Chinese medicine, predicting target genes of Chinese medicine and disease, constructing of a protein interaction network, and conducting GO and KEGG enrichment analysis. Finally, the results of network pharmacology were further validated by molecular docking experiments and cell experiments. Eight active constituents and 14 potential protein targets were screened from RP, including EGFR, JAK2 and SRC. The biological mechanism of RP against CC was analysed by studying the relationship between active components, targets, and enrichment pathways. These findings provide a basis for understanding the clinical application of RP in CC.

## Introduction

Colon cancer (CC) is the second most common cause of cancer death and the third most common cancer in the world^1^. The decline in the incidence and mortality of colon cancer in developed countries may be associated with increased cancer screening in developed countries. However, the rapid rise in mortality rates in developing countries and the fact that more than half of the patients were diagnosed as late-stage significantly increased the medical and economic burden in developing countries^2^. Despite progress in treatment and care in recent years, many treatments are still accompanied by severe adverse reactions and drug resistance, which can reduce the quality of life and increase patient suffering. Therefore, adjuvant therapy and alternative therapies with low toxicity are necessary to improve quality of life and prolong lifetime. Traditional Chinese medicine (TCM), as a critical adjuvant to tumour therapy, can improve the effectiveness of treatment, reduce drug resistance, and prolong survival time^3^. For example, adjuvant treatment with Chinese herbal medicine can reduce the hepatotoxicity of colon cancer patients^4^. Furthermore, alkaloids can inhibit the growth of colon cancer cells through the Wnt/β-catenin signalling pathway^5^. Previous studies have shown that the traditional Chinese medicine Angelica can increase the sensitivity of colon cancer cells to radiotherapy and chemotherapy. In addition, the traditional Chinese medicine ginsenoside Rh2 can reduce the resistance of colon cancer cells to oxaliplatin^6^.

*Radix Puerariae* (RP) is a kind of Chinese medicine that was used for medicine in the Jin Dynasty. RP is a dried root of *Pueraria lobata* (Willd.) Ohwi, which has the effects of reducing heat, stopping diarrhoea, producing body fat, and benefiting^7^. Currently, RP is used in the treatment of many diseases, such as angina, hypertension, diabetes, optic atrophy, and retinitis^8^. RP is also commonly used in the treatment of cancer. In vitro experiments confirmed the anticancer activity of most components of RP, such as puerariae radix isoflavones, by inhibiting the growth of breast cancer cells^9^; puerarin, for example, inhibits metastasis and invasion of liver cancer through PTEN/AKT signalling^10^; and puerarin induces apoptosis of colon cancer cells by increasing the activation of caspase-3^11^. In modern medicine, RP is also often used for the treatment of colon cancer, but the active components and targets of RP treatment for colon cancer are not known. Previous studies have demonstrated the clinical efficacy of traditional Chinese medicine prescriptions containing RP in diabetes through network pharmacology^12^. This study will verify the efficacy of RP on colon cancer through network pharmacology and cell experiments.

Research on TCM and biological networks first appeared in 2007, before the term "network pharmacology" was proposed^13^. The clinical efficacy and mechanism of TCM has been a major difficulty in the study of modern traditional Chinese medicine. Using network pharmacology method can realize the transformation of TCM research from experience medicine to evidence-based medicine, and accelerate the clinical research and application of TCM^14^. TCM Network pharmacology can show the complex relationship between drugs and disease in the form of a network. In the network, the drug target-disease target-pathways network and protein–protein interaction (PPI) network are commonly used to predict the efficacy of drugs to disease. The degree and clustering coefficient are common parameters for evaluating the network. The ADME system refers to the process of drug absorption, distribution, metabolism, and excretion in the body^15–17^. The ADME-based admetSAR method has been proven to be a successful method. We selected the parameters of HIA, Caco-2 and HOB in the admetSAR database to optimize the active ingredient screening strategy.

In the nineteenth century, the receptor theory was proposed to consider the pairing of drug small molecules as ligands and protein macromolecules as receptors to form keys and locks^18^. With the establishment of energy matching and geometric complementary models for ligands and receptors and the development of computer technology, the idea of simulating the interaction between ligands and receptors has been realized, namely, molecular docking analysis. Molecular docking refers to the prediction of binding patterns of ligand small molecules and receptor proteins by continuously optimizing their conformations, positions, amino acid residues and other binding modes when both ligand small molecules and receptor proteins are three-dimensional structures and the minimum energy of ligand small molecules^19^. The method involves screening suitable drugs by scoring affinity.

**Figure 1:**
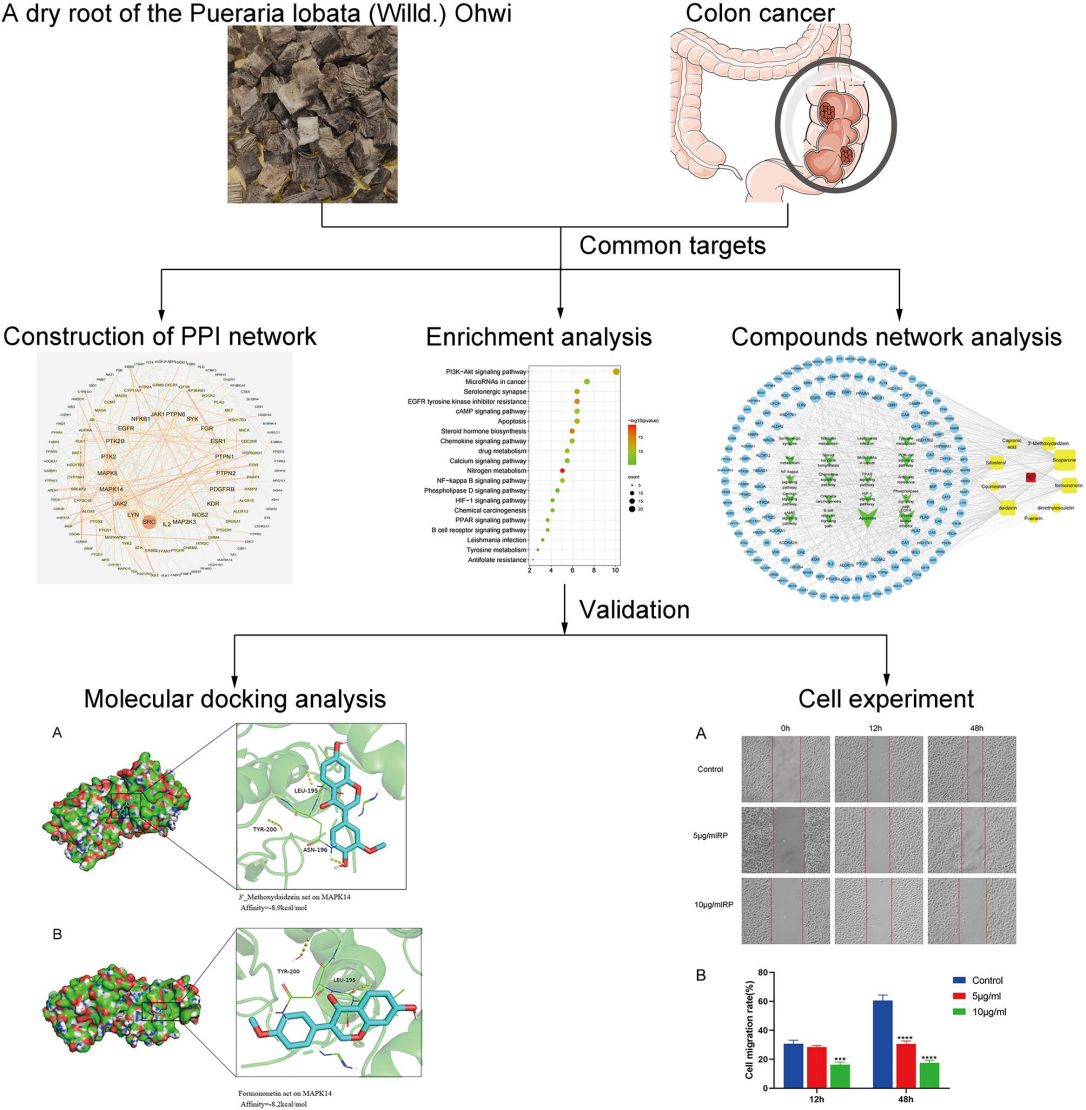
Flow chart for network pharmacology analysis and validation of RP anti-CC.

In this study, the active ingredient of RP and its possible anticancer mechanism were studied using network pharmacology. Key targets and pathways were experimentally verified by molecular docking and in vitro validation. The study flow is shown in Fig. 1.

## Results

### Screening active ingredients of RP

After combining the RP ingredients found in the following databases, there were 40 components, of which 15 components were retrieved from the TCPSP database, 12 components were retrieved from the ETCM database, 10 components were retrieved from the herb database, and 3 components were retrieved from the literature (Supplementary Table S1). The retrieved ingredients were submitted to the admetSAR website (http://lmmd.ecust.edu.cn/admetsar2) for further screening based on the results of human intestinal absorption (HIA), Caco-2, human oral bioavailability (HOB), and plasma protein binding (PPB) (Supplementary Table S2). The results showed that there were 9 components with good absorption and distribution properties, and their chemical constituents were mainly isoflavone, coumarins, and alkaloids (Table 1). Although the Caco-2 and HOB of puerarin predicted by admetSAR were lower, it has an important biological role in previous studies, so it was temporarily retained for further study.

**Table 1:** Partial ADME values and chemical component of the 9 post-screening components.

Components	HIA	Caco-2	HOB	PPB (100%)	Chemical component
Formononetin	+ 0.9911	+ 0.9313	+ 0.5714	1.138	Isoflavones
Daidzein	+ 0.9893	+ 0.9313	+ 0.5714	0.831	Isoflavones
Scoparone	+ 0.9916	+ 0.8389	+ 0.7143	0.986	Coumarins
3′-Methoxydaidzein	+ 0.9911	+ 0.886	+ 0.6	1.055	Isoflavones
Sitosterol	+ 0.993	+ 0.5385	+ 0.5286	1.124	Alkaloids
Dimethylesculetin	+ 0.9812	+ 0.5962	+ 0.6143	1.144	Coumarins
Coumestan	+ 0.9785	+ 0.5843	+ 0.7429	0.91	Coumarins
Capronic acid	+ 0.8417	+ 0.8296	+ 0.7429	0.232	Acid

### Intersection of related targets

**Figure 2:**
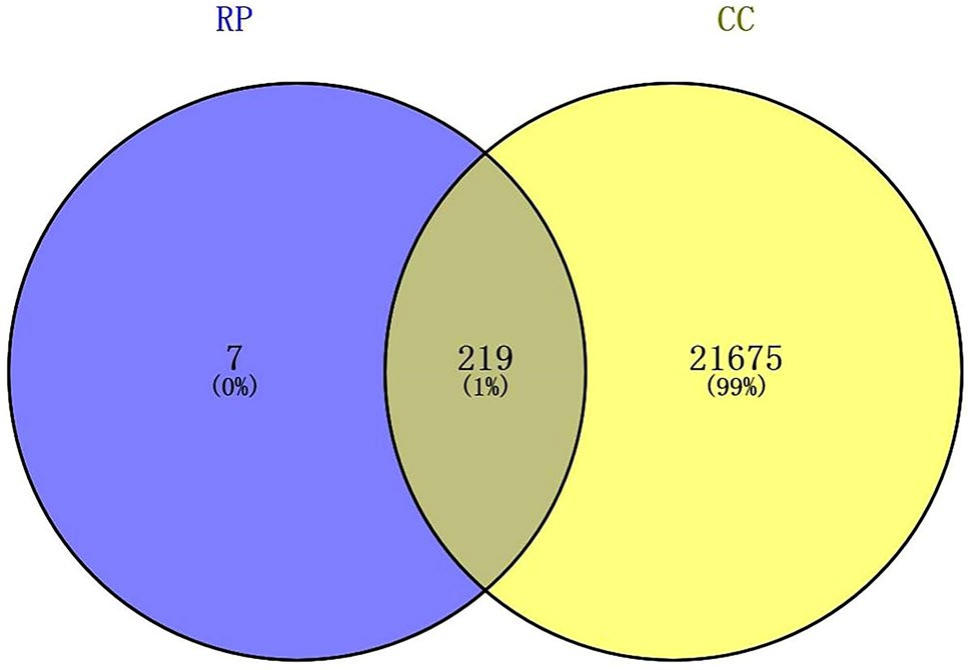
Venn diagram of RP and colon cancer, with 201 overlapping targets.

SwissTargetPrediction is a network tool designed to predict the most likely protein targets in small molecules by reverse screening based on similarity principles. Submit the SMILES descriptions of puerarin and the screened active ingredients to the SwissTargetPrediction database and output the results with a similarity probability > 0 to the target (Supplementary Table S3). By combining the targets of active ingredients and deleting duplicates, we obtained 226 targets related to RP. A total of 21,894 colon cancer-related targets were obtained from GeneCards and OMIM. Then, we inputted RP and CC targets to Venny 2.1, and the results showed that there were 219 common gene targets between RP and CC (Supplementary Table S4) (Fig. 2).

### Analysis of target PPI network

**Figure 3:**
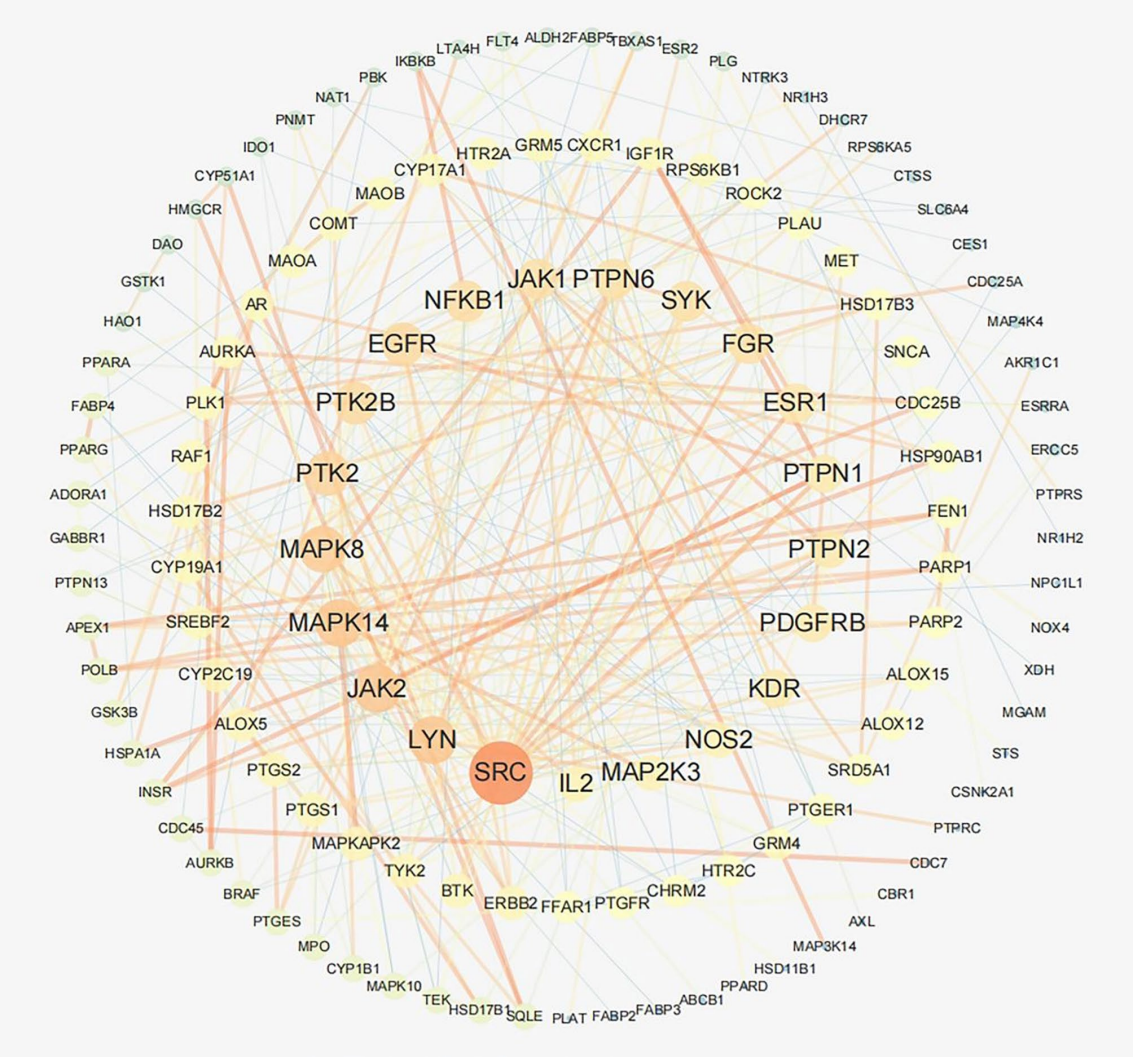
Visualization analysis of the target PPI network.

The STRING database was used to show the link between proteins participating in specific biological functions. We entered common targets into the "Multiple proteins" of the STRING database, selected the highest confidence level and hid the disconnected nodes in the network to obtain the interaction between them. Then, the results of TSV format were imported into Cytoscape, and 104 nodes and 218 edges were displayed (Fig. 3). After calculating and visualizing the target degree values in the PPI network, the genes with higher degrees were SRC, LYN, JAK2, MAPK14, MAPK8, PTK2, PTK2B, EGFR, NFKB1, JAK1, PTPN6, SYK, FGR and ESR1.

### GO and KEGG analysis

**Figure 4:**
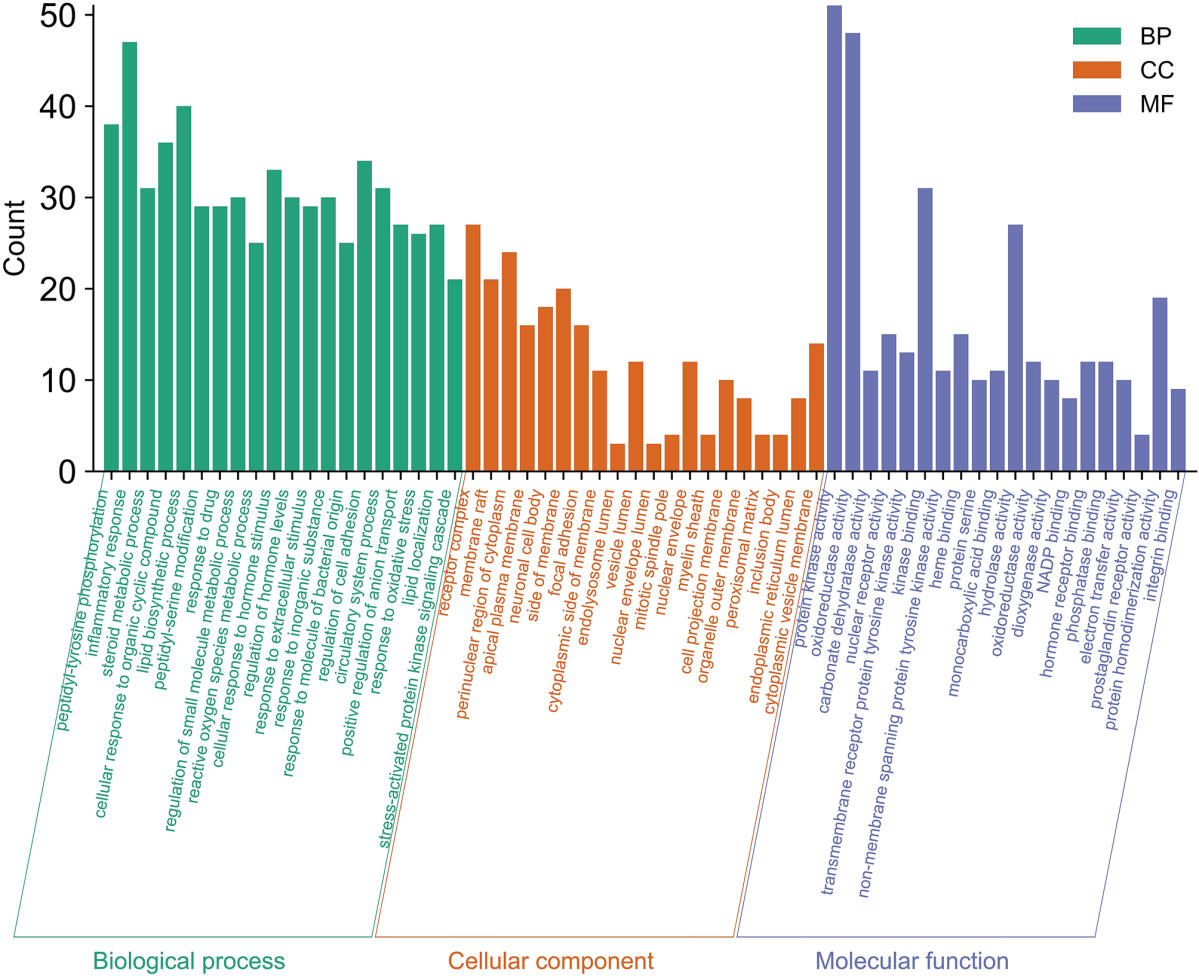
GO enrichment analysis of 219 targets related to RP and CC common targets. The *x* axis represents GO terms, and the *y* axis represents the number of genes enriched in each GO term (p < 0.01).

**Figure 5:**
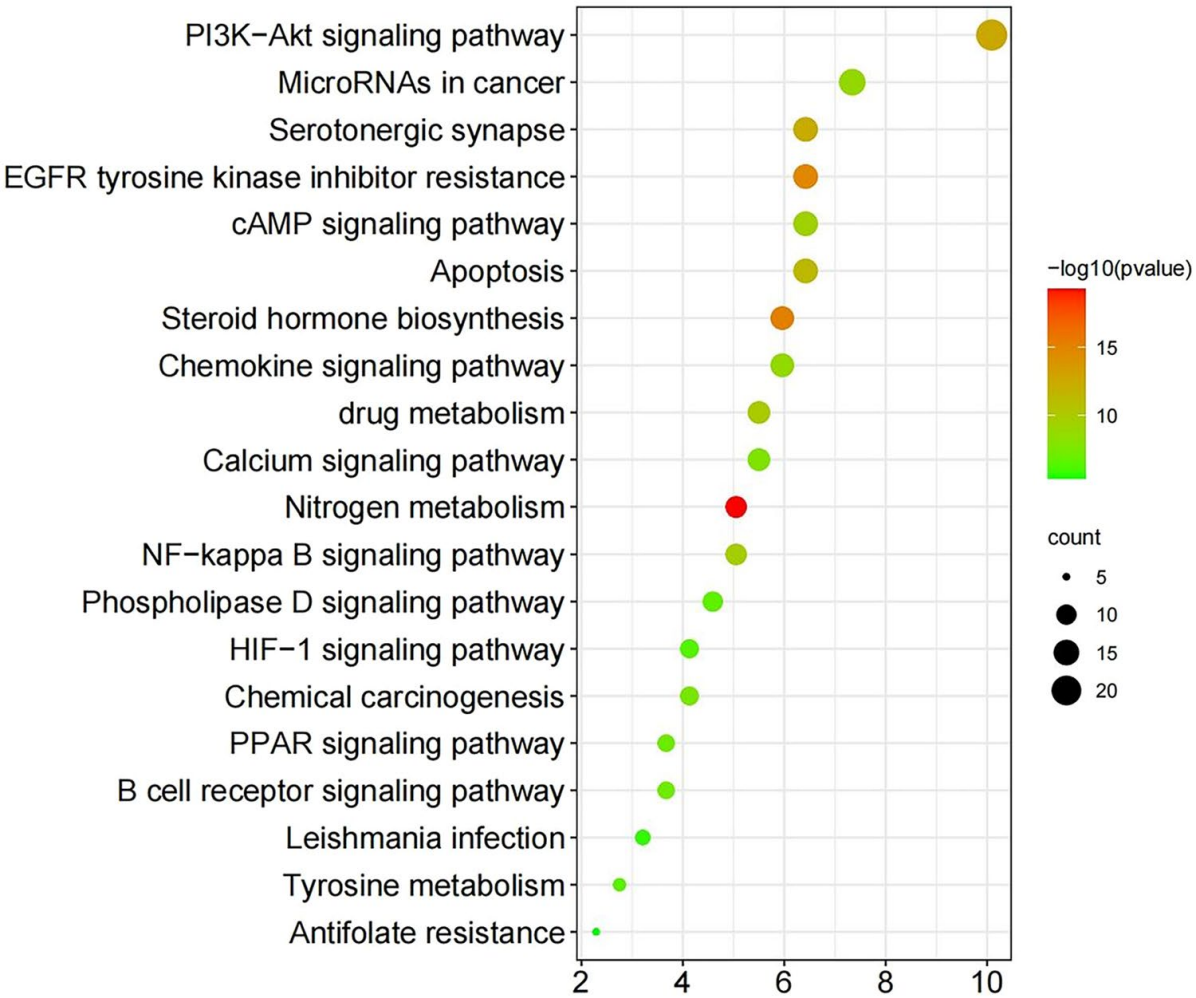
KEGG pathway analysis. The *X*-axis refers to the number of enriched targets in a pathway as a percentage of total targets, and the *Y*-axis refers to the enrichment pathways. The size of the points depends on the number of targets enriched in a pathway. The colour of the points depends on − log10(p value). The darker the colour is, the more significant the difference.

We used Metascape for GO function and KEGG pathway analysis to further understand the mechanism of RP on CC and set the significance cutoff to P < 0.01. GO function results included three parts: biological process (BP), cell component (CC) and molecular function (MF). In BP GO terms, peptidyl-tyrosine phosphorylation, regulation of cell adhesion, responses to drug, etc. may be associated with tumour regulation. CC terms were mainly enriched in the perinuclear region of the cytoplasm, membrane raft, focal adhesion, etc. In MF GO terms, protein kinase activity, kinase binding, and transmembrane receptor protein tyrosine kinase activity may be associated with tumours (Fig. 4). In KEGG enrichment pathways, EGFR tyrosine kinase inhibitor resistance, the PI3K-Akt signalling pathway, apoptosis, and the NF-kappa B signalling pathway were involved in apoptosis and cancer regulation (Fig. 5). These pathways may be critical in the treatment of CC. The results of KEGG analysis were compared with the PPI network, and 7 targets with higher degrees were randomly selected for receptor proteins in molecular docking, including SRC, JAK2, MAPK14, EGFR, NFKB1, ESR1, and IL2.

### Compounds target network analysis

**Figure 6:**
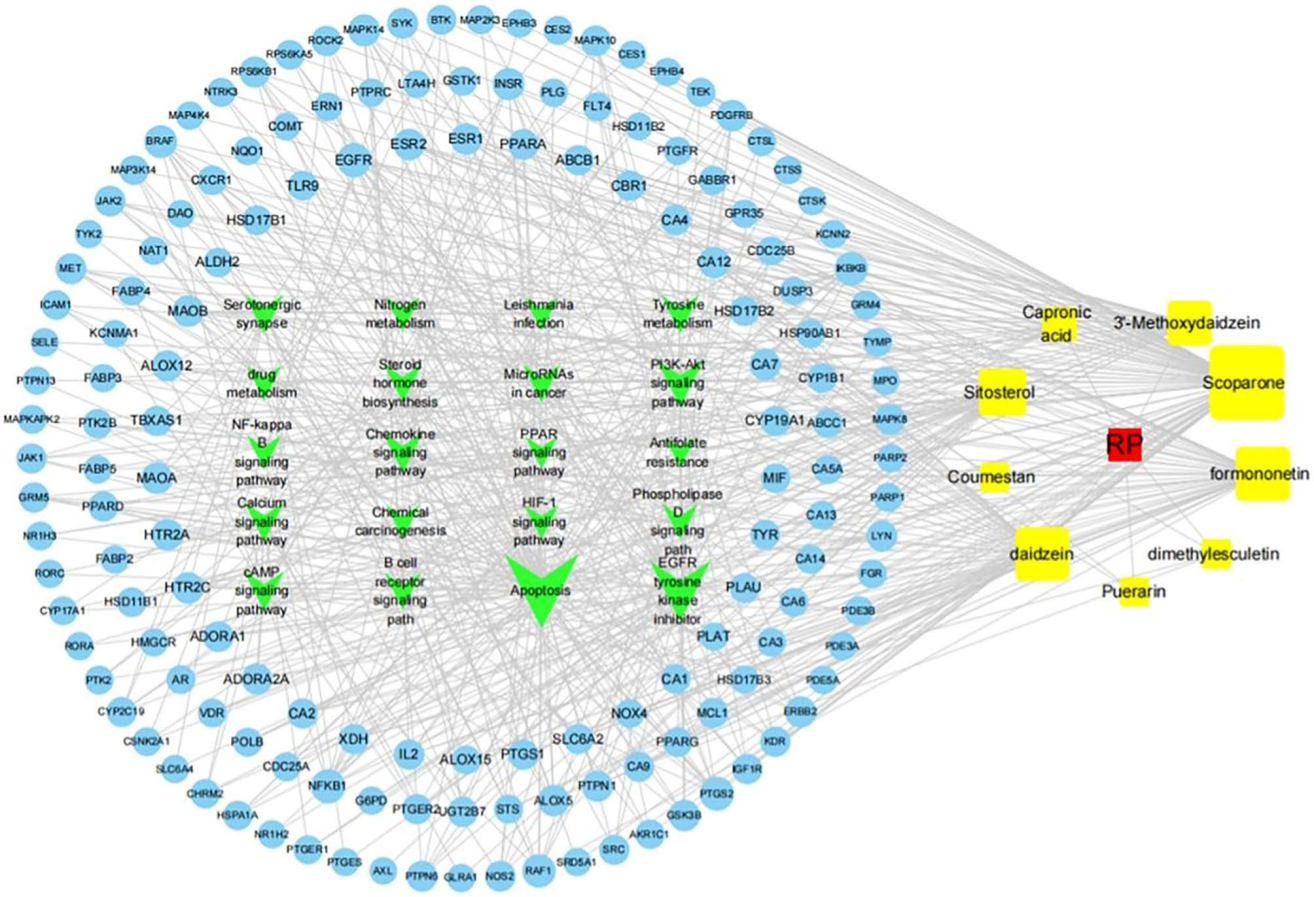
RP component-target-pathway network analysis. The red node refers to the drug; the green node represents pathways; the blue nodes represent targets; and the yellow nodes indicate components. The edges indicate their interactions.

The top 20 pathways with the largest number of genes were chosen to construct an RP component-target-pathway network with 183 nodes and 626 edges (Supplementary Table S5) (Fig. 6). The red node refers to the drug; the green node represents pathways; the blue nodes represent targets; and the yellow nodes indicate components. The edges indicate their interactions. Each compound interacts with multiple targets in the graph, suggesting that the effect of RP on CC may be a synergistic effect of multiple targets. According to the degree of topological parameters of the network, five high-level components were selected as ligand molecules in molecular docking. Of the five ingredients, three isoflavone components, namely, formononetin, daidzein, and 3′-methoxydaidzein; one alkaloid component, namely, sitosterol; and one coumarin component, namely, scoparone.

### Molecular docking analysis

**Figure 7:**
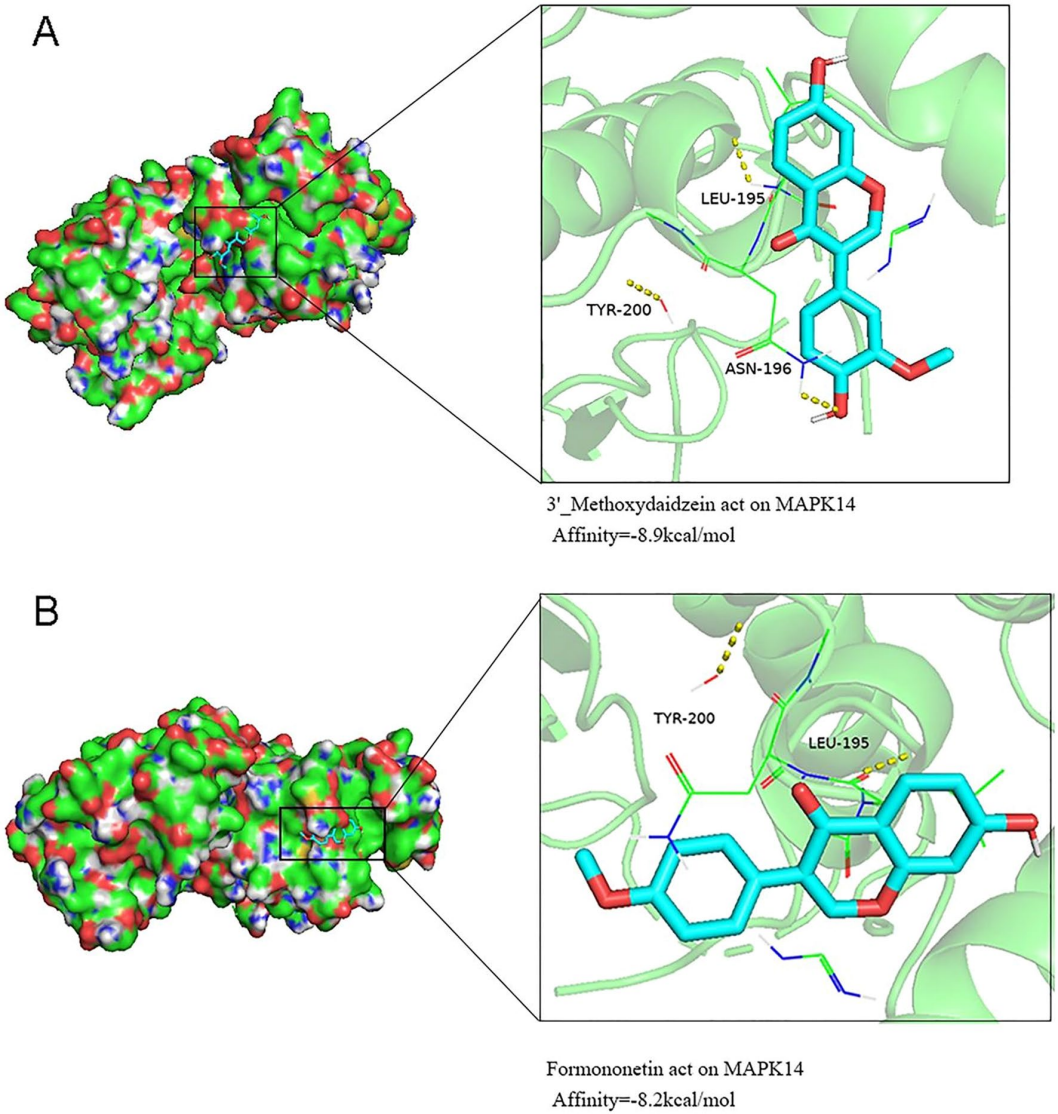
The docking complexes of ligand and receptor proteins and their binding residues are shown using PYMOL software. (**A**) The interaction between MAPK14 and 3′-methoxydaidzein occurs through the amino acid residues LEU-195, TYR-200, and ASN-196. (**B**) The interaction between MAPK14 and formononetin occurs through the amino acid residues LEU-195 and TYR-200.

Based on the results of the interaction network, we selected the targets and ingredients with higher degrees for molecular docking experiments. The PDB entry numbers of the target structures selected from the PDB database were EGFR (5UG9), JAK2 (3UGC), MAPK14 (2FST), NFKB1 (1SVC), ESR1 (3CBP), IL2 (4NEJ), and SRC (1O43). We conducted molecular docking between receptor proteins and ligand molecules through AutoDockTools 1.5.6. The result of AutoDockTools was output in the form of an affinity score, which is the core parameter of AutoDockTools (Table 2). The lower the affinity score is, the better the binding effect. PYMOL software visualized the docking complexes and binding residues of 3′-methoxydaidzein and formononetin ligand molecules with MAPK14 receptor proteins (Fig. 7).

**Table 2:** Affinity score of the screened 5 ligand molecules to 7 receptor proteins.

Compound	Affinity score (kcal/mol)						
	EGFR	JAK2	MAPK14	NFKB1	ESR1	IL2	SRC
Scoparone	− 6	− 7	− 6.6	− 5.5	− 7.1	− 5.8	− 5.1
Sitosterol	− 6.2	− 8.1	− 7.5	− 6.5	− 7.2	− 7.2	− 6.5
formononetin	− 7.5	− 8.5	− 8.2	− 6.6	− 8.7	− 6.6	− 6.2
daidzein	− 7.5	− 9.5	− 7.8	− 6.8	− 8.5	− 6.4	− 6.4
3′-Methoxydaidzein	− 7.6	− 8.7	− 8.9	− 6.9	− 8.8	− 6.4	− 6.4

### RP inhibits proliferation of colon cancer cells

**Figure 8:**
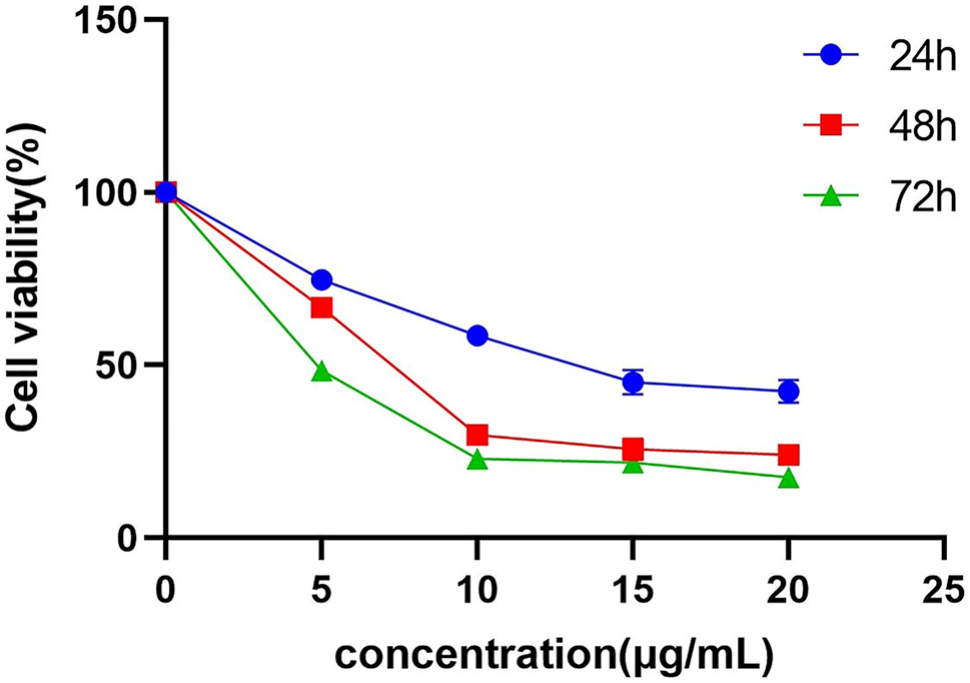
Through the CCK-8 experiment, RP inhibited the proliferation of CC cells. SW480 cells were treated with RP at different concentrations for 24, 48, and 72 h. The data are expressed as the mean ± SD of three separate experiments.

The anticancer effect of RP at different concentrations (0, 5, 10, 15, and 20 μg/mL) on SW480 cells after 24, 48 and 72 h were verified by CCK-8 experiments. The proliferation of SW480 cells treated with different concentrations of RP decreased in a dose- and time-dependent manner (Fig. 8). The proliferation ability of SW480 cells decreased linearly under low concentrations of RP but did not change much under high concentrations of RP. The IC50 values 24, 48 and 72 h after RP treatment were 14.9, 9.8, and 8.0 μg/mL in SW480 cells, respectively.

### RP inhibits migration of colon cancer cells

**Figure 9:**
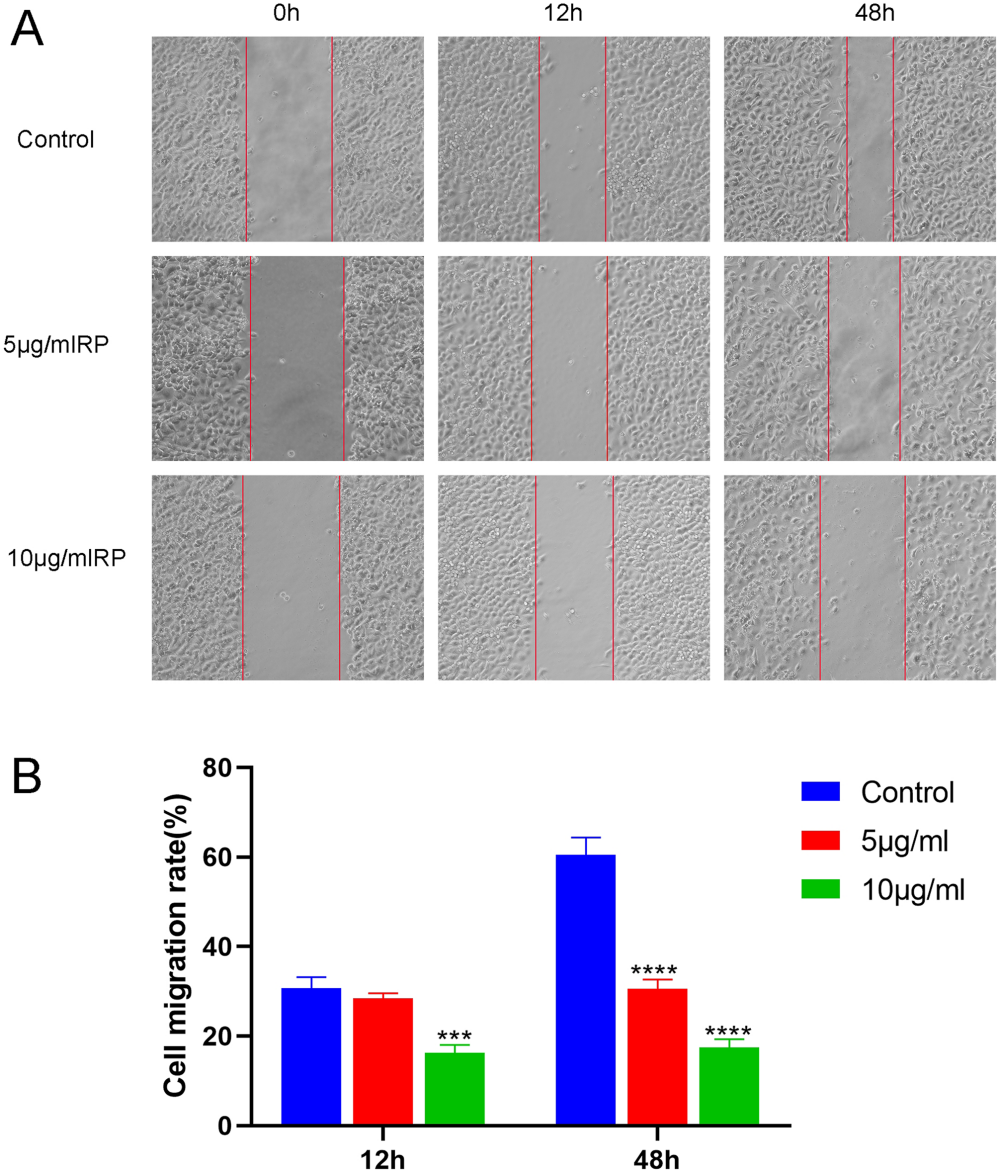
RP significantly inhibited the migration of SW480 cells. The effect of RP (0, 5, 10 μg/mL) on SW480 cell migration activity was examined under an inverted microscope to evaluate cell migration (40 ×). (**A**) Images during cell migration in scratch experiments. (**B**) The average scratch cell mobility at 12 h and 48 h after 5 μg/mL and 10 μg/mL treatment. ***p < 0.001, ****p < 0.0001 compared with the control (0 μg/mL).

The effect of RP on SW480 cell migration was studied by the scratch test (Fig. 9). As shown in the figure, at 12 h, the cell scratches of the three groups were reduced by different treatment methods, and the reduction range of the control group was the largest; at 48 h, the control group was further reduced by a large margin, but there was no significant change in the two groups after treatment with RP medium. The results showed that the effect of RP on SW480 cell migration was more significant over time than that of the control group. This result suggested that RP could inhibit the invasion and migration activity of SW480 cells.

## Discussion

Cancer is a common disease with a complex pathogenesis characterized by abnormal cell growth and uncontrolled division. With the emergence of problems such as side effects and drug resistance to anticancer drugs, many researchers have aimed to find natural anticancer drugs with fewer side effects. As a natural product with abundant resources, TCM has attracted increasing attention because of its unique advantages in reducing the side effects of anticancer drugs, prolonging the survival rate of patients, and improving the life of patients^20^. Because of the complex composition of natural products, the development and use of traditional Chinese medicine also face new problems. Therefore, the screening method based on ADME characteristics has been widely recognized in the development and application of traditional Chinese medicine^21^.

RP is a Chinese herb with anticancer, anti-inflammatory, anti-oxidant and cardiovascular protective properties. RP is mainly used in the treatment of cancer, endocrine disease, cardiovascular disease, and neurodegeneration. The results of RP component screening showed that the activity of isoflavones was the strongest, followed by alkaloids and coumarins. Previous studies on the anticancer effects of RP have focused primarily on the role of puerarin, and few studies have examined other active components of RP^11,22–24^. Studies on formononetin, daidzein and other RP components show that they can also inhibit growth and induce apoptosis in human colon cancer cells. Formononetin in vivo experiments showed that it can inhibit the growth and proliferation of human colon cancer cells and reduce the invasiveness and vascular endothelial growth factor (VEGF) expression level in tumour tissues^25^. The genotoxic and cytotoxic effects of daidzein on human colon adenocarcinoma cells are dose-dependent^26^. These RP active ingredients were successfully screened by ADME parameters.

PPI network analysis of common targets for RP and colon cancer showed that 14 targets had a high degree of SRC, LYN, JAK2, MAPK14, MAPK8, PTK2, PTK2B, EGFR, NFKB1, JAK1, PTPN6, SYK, FGR and ESR1. In molecular docking experiments, EGFR and 3'-methoxydaidzein showed high affinity, indicating that EGFR may be one of the key targets of RP in the treatment of CC.

Epidermal growth factor receptor (EGFR) is a cancer-promoting tumour marker that regulates the homeostasis and growth of epithelial tissues and cells. EGFR is also considered to be associated with the development of tumour resistance due to the occurrence of point mutations and amplification following the use of anticancer drugs^27^. EGFR is a member of receptor tyrosine kinase (RTK), a family of proteins that needs to form heterodimers to function. The upregulation of EGFR activity is mediated by common truncated and mutated extracellular domains, and upregulated EGFR overactivates downstream procancer signalling pathways, including the AKT-PI3K-mTOR and RAS-RAF-MEK-ERK MAPK pathways^28^. These pathways then activate a variety of biological effects that are beneficial to the proliferation of cancer cells, causing cancer to occur and develop. This evidence suggests that RP may play a role in the treatment of colon cancer by downregulating EGFR protein expression.

In the GO functional analysis, we screened the first 20 terms out of the three parts BP, CC and MF based on a P value < 0.01. In BP GO terms, peptidyl-tyrosine phosphorylation, response to drug, CC terms membrane raft, and in MF GO terms, protein kinase activity, kinase binding, and transmembrane receptor protein tyrosine kinase activity may be associated with tumours. Then, we screened the first 20 pathways obtained from KEGG enrichment analysis according to the criteria of a P value < 0.01. The pathway with the most enriched genes was the PI3K-Akt signalling pathway, with 22 targets enriched in this pathway, including EGFR, HSP90AB1, IL2, JAK2, NFKB1, MAPK14, SRC, etc. Phosphatidylinositol 3′-kinase (PI3K)-Akt is a signalling pathway that regulates the basic functions of cells, such as transcriptional translation and growth and proliferation, and can be activated by toxic damage or cell stimulation^29,30^. PI3K catalyses the formation of phosphatidylinositol 3,4,5-triphosphate (PIP3) on the cell membrane, and PIP3 acts as a second messenger to activate Akt. Finally, Akt regulates the key processes of cell growth and development by phosphorylating proteins involved in cell synthesis, metabolism, the cell cycle and apoptosis. RP may play an anti-CC role by regulating proteins enriched in the PI3K-Akt signalling pathway.

The use of TCM has a long history, but its mechanism and target are not clear. Molecular docking experiments can further verify the accuracy of network pharmacology results by simulating the interaction between active ingredients and targets, and replace some exploratory experiments. Therefore, network pharmacology integration molecular docking experiment can improve the efficiency of TCM research. Both network pharmacology and molecular docking experiments are bioinformatics studies, which cannot directly reflect the dose–effect relationship, so the combination of the two has certain limitations^31^.

## Methods

### Screening of active ingredient

The components of RP were obtained from the Traditional Chinese Medicine Systems Pharmacology Database and Analysis Platform^32^ (https://tcmspw.com/tcmsp.php), the Encyclopedia of Traditional Chinese Medicine^33^ (http://www.tcmip.cn/ETCM/index.php/Home/), Herb^34^ (http://herb.ac.cn/), and the literature. Then, canonical SMILES of these ingredients was obtained from NCBI PubChem^35^ (https://pubchem.ncbi.nlm.nih.gov). Finally, the SMILES descriptions were input into admetSAR (http://lmmd.ecust.edu.cn/admetsar2/) to screen active components of RP according to HIA, Caco-2 and HOB. AdmetSAR database can be divided into positive and negative results by various machine learning algorithms and molecular fingerprints^36^. The positive value indicates that the active ingredient has good ADME performance. We retained active ingredients with positive HIA, Caco-2 and HOB parameters. Ingredients that did not meet the screening requirements but were significantly bioactive in previous studies were retained.

### Acquisition of gene targets

The screened active ingredient smiles were uploaded to Swiss Target Prediction^37^ (http://www.swisstargetprediction.ch/) to predict the target, the species was set as Homo sapiens, and the result of probability > 0.1 was selected. At the same time, colon cancer-associated target genes were obtained from GeneCards^38^ (https://www.genecards.org/) and Online Mendelian Inheritance in Man (https://omim.org/)^39^, and the results of colon cancer-target genes from both databases were combined. Then, we used Venny 2.1 (http://bioinfogp.cnb.csic.es/tools/venny/index.html) to obtain the gene target intersection of RP and colon cancer.

### Construction of protein–protein interaction (PPI) network

Gene targets overlapping with RP and CC were input into the STRING database^40^ (https://string-db.org) to obtain the PPI network. The parameter was set to Homo sapiens, and the interactive score was set to the highest credibility. Then, the target intersection file was imported into Cytoscape 3.7.1 for visual analysis of the PPI network. We used the network analysis tools in Cytoscape to analyse the degree values of nodes and edges in the network. The higher the degree value is, the larger the node size and the brighter the edge colour in the network.

### GO and KEGG enrichment analysis

We submitted the intersecting target genes to Metascape^41^ (http://metascape.org), selected “*H. sapiens*” as the input species, set a p value < 0.01, and then, we conducted Gene Ontology (GO) terms and Kyoto Encyclopedia of Genes and Genomes (KEGG) pathway enrichment analysis. The top 20 higher-score GO enrichment or KEGG pathways were analysed^42^.

### Construction of interactive network

To understand the relationship between components, targets, and enrichment pathways, we used Cytoscape 3.7.1^43^ to conduct visual analysis and construct an interactive network. First, we established a file in which the active ingredient and target correspond to each other, and the target and pathway correspond to each other. We then created an attribute file that named the active ingredient, target, and pathway as different types. Finally, we input the above two files into the Cytoscape 3.7.139 software. We used the network analysis tool in this software to analyse the network degree value so that the nodes in the network showed different sizes. A larger node represents a closer connection between that node and other nodes in the network.

### Molecular docking

According to the interactive network results of RP components and targets, molecular docking experiments were carried out at the nodes to a larger degree. The 2D SDF structure files of components were obtained by NCBI PubChem, and the SDF files were input into Chem3D 20.0 software to minimize energy. The targets were entered into the UniProt^44^ (https://www.uniprot.org) database and filtered by reviewed and human organisms, and then, the higher resolution, single chain and X-ray pathway were selected according to the structural information. The selected crystal structures were downloaded from the Protein Data Bank database (https://www.rcsb.org/) in pdb format^45,46^. Then, the targets were deliganded and dewatered by PYMOL 2.3.4 software, hydrogenated and charge balanced by AutoDockTools 1.5.6 software, and treated by the Grid Option tool. Finally, targets and components were converted into pdbqt format. We used AutoDockTools to evaluate the affinity of components and target proteins by the same docking method. The docking complexes of ligand and receptor proteins and their binding residues were visualized by PYMOL software^47,48^.

### Cell culture

The human colon cancer cell line SW480 used in this study was obtained from the cell library of the Chinese Academy of Sciences (Shanghai, China). The cells were preserved in Dulbecco’s modified Eagle’s medium (DMEM) containing 1% penicillin/streptomycin (Corning) and 10% foetal bovine serum (Gibco).

### CCK-8 assay

The cell viability of CC cells was determined by Cell Counting Kit-8 (CCK-8) assay. SW480 cells were inoculated into 96-well plates at a density of 4 × 10^4^/mL. After overnight culture, the cells were treated with RP at 0, 5, 10, 15, and 20 μg/mL in DMEM for 24, 48, and 72 h. The cells were then incubated with Cell Counting Kit-8 at 37 °C for 2.5 h in a 5% carbon dioxide incubator, and their absorbance values at 450 nm were measured. The results were analysed and plotted with GraphPad Prism 8.0 software.

### Scratch test

SW480 cells in the exponential growth phase were inoculated in 6-well plates (5 × 10^5^ cells/well). When the cells reached a state of fusion into a monolayer, 3 uniform lines were drawn vertically along the plate with a pipette aspirator with a sterile diameter of 2 mm. The cells were then incubated for 0, 12, and 48 h with drug-free medium (serum-free medium) and drug-containing medium (serum-free medium containing 5 and 10 μg/mL RP). Each group was repeated three times. Photographs were taken with an inverted microscope (Leica, Germany) to observe the migration rate of cells in each group.

### Statistical analysis

All data were measured in three separate experiments and are expressed as the mean ± SD. The differences between the treatment and control groups were analysed by GraphPad Prism 8.0 (GraphPad Software Inc., USA) one-way analysis of variance (ANOVA). p < 0.05 indicated statistically significant differences.

## Supplementary Information

Supplementary Tables.

## Data Availability

Supplementary files are provided for drug targets, ADME screening results, common targets for drugs and diseases, and RP component-target-pathway network results.
