# Supplementary material for: Identification of the potential mechanism of Radix pueraria in colon cancer based on network pharmacology
Source: Sci Rep. 2022 Mar 8;12:3765. doi: 10.1038/s41598-022-07815-y (PMC8904787; doi:10.1038/s41598-022-07815-y)
Supplement: Supplementary file 1 — Supplementary Tables. [file 41598_2022_7815_MOESM1_ESM.pdf]

Supplementary Table S1: RP components from TCMSP, ETCM, herbs, literature and CAS numbers for these components.

Components	CASnumber	Source
formononetin	485-72-3	TCMSP
daidzein	486-66-8	TCMSP
Ononin	486-62-4	TCMSP
Docosanoate	2636-16-0	TCMSP
LUPENONE	1617-70-5	TCMSP
genistein	446-72-0	TCMSP
lignoceric acid	557-59-5	TCMSP
scoparone	120-08-1	TCMSP
(R)-Allantoin	885215-81-6	TCMSP
3'-Methoxydaidzein	21913-98-4	TCMSP
Daidzein-4,7-diglucoside	53681-67-7	TCMSP
Soyasapogenol B	595-15-3	TCMSP
puerarin	3681-99-0	TCMSP
7,8,4'-Trihydroxyisoflavone	75187-63-2	TCMSP
daidzin	552-66-9	TCMSP
Sitosterol	68555-08-8	ETCM
Dimethylesculetin	120-08-1	ETCM
Arachic Acid	506-30-9	ETCM
Arachidic Acid	506-30-9	ETCM
Daucosterol	474-58-8	ETCM
Caproic Acid	142-62-1	ETCM
Eleutheroside A	474-58-8	ETCM
Formononetin-7-Glucoside	18524-03-3	ETCM
Coumestrol	479-13-0	ETCM
Genistein 7-Glucoside	529-59-9	ETCM
Puerarin-Xyloside I	114240-18-5	ETCM
Tuberosin	41347-45-9	ETCM
1-formyl-beta-carboline	20127-63-3	herb
4'-methoxypuerarin	92117-94-7	herb
5-methylhydantoin	616-03-5	herb
coumingidine	434898-80-3	herb
dauricine	524-17-4	herb
methyl-p-hydroxycinnamate	3901-07-3	herb
puerarin-xyloside	114240-18-5	herb
puerarol	158020-56-5	herb
Robustadial A	88197-30-2	herb
Scopine	498-45-3	herb
Isoliquiritigenin	961-29-5	Literature
d-mannitol	69-65-8	Literature
coumestan	479-12-9	Literature

Supplementary Table S2: Results of HIA, Caco-2, HOB, and PPB for RP components from admetSAR.

Components	HIA	Caco-2	HOB	PPB（100%）	Source
formononetin	+0.9911	+0.9313	+0.5714	1.138	TCMSP
daidzein	+0.9893	+0.9313	+0.5714	0.831	TCMSP
Ononin	-0.4104	-0.8314	-0.5714	0.876	TCMSP
Docosanoate	+0.8476	-0.5365	-0.6714	1.027	TCMSP
LUPENONE	+0.9914	-0.5206	-0.5571	1.133	TCMSP
genistein	+0.9881	+0.9525	-0.6	0.88	TCMSP
lignoceric acid	+0.9826	-0.7491	-0.5857	1.032	TCMSP
scoparone	+0.9916	+0.8389	+0.7143	0.986	TCMSP
(R)-Allantoin	+0.9595	-0.8144	+0.7429	0.376	TCMSP
3'-Methoxydaidzein	+0.9911	+0.886	+0.6	1.055	TCMSP
Daidzein-4,7-diglucoside	-0.5168	-0.9071	-0.5857	0.883	TCMSP
Soyasapogenol B	+0.9884	+0.5326	-0.5143	0.826	TCMSP
puerarin	+0.8959	-0.9373	-0.7	0.859	TCMSP
7,8,4'-Trihydroxyisoflavone	+0.9852	+0.7064	-0.6143	0.849	TCMSP
daidzin	-0.4589	-0.9373	-0.7143	0.766	TCMSP
Sitosterol	+0.993	+0.5385	+0.5286	1.124	ETCM
Dimethylesculetin	+0.9812	+0.5962	+0.6143	1.144	ETCM
Arachic Acid	+0.8417	+0.5331	-0.6714	0.871	ETCM
Arachidic Acid	+0.8417	+0.7107	-0.6714	0.851	ETCM
Daucosterol	-0.6476	-0.8381	-0.7143	1.035	ETCM
Caproic Acid	+0.8417	+0.8296	+0.7429	0.232	ETCM
Eleutheroside A	+0.9266	-0.766	-0.7143	1.023	ETCM
Formononetin-7-Glucoside	-0.4104	-0.8314	-0.5714	0.888	ETCM
Coumestrol	+0.9856	-0.5364	+0.5571	0.854	ETCM
Genistein 7-Glucoside	+0.9348	-0.7971	-0.7429	0.808	ETCM
Puerarin-Xyloside I	+0.9263	-0.8661	-0.7429	0.666	ETCM
Tuberosin	+0.9881	+0.5673	-0.6571	1.079	ETCM
1-formyl-beta-carboline	+0.9928	-0.5643	+0.5571	0.729	herb
4'-methoxypuerarin	+0.9742	-0.8961	-0.6286	0.842	herb
5-methylhydantoin	+0.9763	-0.7813	+0.7857	0.486	herb
coumingidine	+0.9938	-0.6253	-0.7	1.208	herb
dauricine	+0.9864	-0.7621	-0.5286	1.004	herb
methyl-p-hydroxycinnamate	+0.9868	+0.9033	-0.7286	0.704	herb
puerarin-xyloside	+0.9263	-0.866	-0.7429	0.666	herb
puerarol	+0.9839	-0.7122	-0.5571	0.999	herb
robustadial a	+0.9831	+0.6544	-0.5286	1.095	herb

Scopine	+0.9547	+0.6525	-0.5	0.554	herb
Isoliquiritigenin	+0.9803	+0.9313	-0.5286	0.62	Literature
d-mannitol	+0.8011	-0.9372	-0.7571	0.064	Literature
coumestan	+0.9785	+0.5843	+0.7429	0.91	Literature

Supplementary Table S3: The probability of the active ingredient predicted by the Swiss Target Prediction database is greater than 0 for relevant target informations.

Constituent	Target	Uniprot ID	Probability
3'-Methoxydaidzein	TYR	P14679	0.196657136
3'-Methoxydaidzein	MIF	P14174	0.196657136
3'-Methoxydaidzein	CYP19A1	P11511	0.180252494
3'-Methoxydaidzein	CA7	P43166	0.180252494
3'-Methoxydaidzein	HSD17B2	P37059	0.180252494
3'-Methoxydaidzein	CA12	O43570	0.180252494
3'-Methoxydaidzein	CA4	P22748	0.180252494
3'-Methoxydaidzein	CBR1	P16152	0.180252494
3'-Methoxydaidzein	ABCB1	P08183	0.163737226
3'-Methoxydaidzein	PPARA	Q07869	0.147256737
3'-Methoxydaidzein	ESR1	P03372	0.130791955
3'-Methoxydaidzein	ESR2	Q92731	0.130791955
3'-Methoxydaidzein	EGFR	P00533	0.122581769
3'-Methoxydaidzein	PTPRS	Q13332	0.122581769
3'-Methoxydaidzein	TLR9	Q9NR96	0.114337559
3'-Methoxydaidzein	HSD17B1	P14061	0.114337559
3'-Methoxydaidzein	ALDH2	P05091	0.106099949
3'-Methoxydaidzein	MAOB	P27338	0.106099949
3'-Methoxydaidzein	ALOX12	P18054	0.106099949
3'-Methoxydaidzein	TBXAS1	P24557	0.106099949
3'-Methoxydaidzein	MAOA	P21397	0.106099949
3'-Methoxydaidzein	MGAM	O43451	0.106099949
3'-Methoxydaidzein	HTR2A	P28223	0.106099949
3'-Methoxydaidzein	HTR2C	P28335	0.106099949
3'-Methoxydaidzein	ADORA1	P30542	0.106099949
3'-Methoxydaidzein	ADORA2A	P29274	0.106099949
3'-Methoxydaidzein	ESRRA	P11474	0.106099949
3'-Methoxydaidzein	ESRRB	O95718	0.106099949
3'-Methoxydaidzein	ABCG2	Q9UNQ0	0.106099949
3'-Methoxydaidzein	CA2	P00918	0.106099949
3'-Methoxydaidzein	XDH	P47989	0.106099949
3'-Methoxydaidzein	PTGS1	P23219	0.097874534

3'-Methoxydaidzein	SLC6A2	P23975	0.097874534
3'-Methoxydaidzein	PFKFB3	Q16875	0.097874534
3'-Methoxydaidzein	IL2	P60568	0.097874534
3'-Methoxydaidzein	NOX4	Q9NPH5	0.097874534
3'-Methoxydaidzein	ALOX15	P16050	0.097874534
3'-Methoxydaidzein	CA1	P00915	0.097874534
3'-Methoxydaidzein	PON1	P27169	0.097874534
3'-Methoxydaidzein	ACHE	P22303	0.097874534
3'-Methoxydaidzein	PLAT	P00750	0.097874534
3'-Methoxydaidzein	F10	P00742	0.097874534
3'-Methoxydaidzein	PLAU	P00749	0.097874534
3'-Methoxydaidzein	CDC7	O00311	0.097874534
3'-Methoxydaidzein	PTPN1	P18031	0.097874534
3'-Methoxydaidzein	ABCC1	P33527	0.097874534
3'-Methoxydaidzein	CYP1B1	Q16678	0.097874534
3'-Methoxydaidzein	STS	P08842	0.097874534
3'-Methoxydaidzein	HSP90AB1	P08238	0.097874534
3'-Methoxydaidzein	DUSP3	P51452	0.097874534
3'-Methoxydaidzein	CDC25B	P30305	0.097874534
3'-Methoxydaidzein	MCL1	Q07820	0.097874534
Coumestan	NFKB1	P19838	0.098947479
Coumestan	ACHE	P22303	0.071715932
Coumestan	ESR1	P03372	0.062621967
Coumestan	ESR2	Q92731	0.062621967
Coumestan	CA2	P00918	0.053556076
Coumestan	CA7	P43166	0.053556076
Coumestan	CA1	P00915	0.053556076
Coumestan	CA3	P07451	0.053556076
Coumestan	CA6	P23280	0.053556076
Coumestan	CA12	O43570	0.053556076
Coumestan	CA14	Q9ULX7	0.053556076
Coumestan	CA9	Q16790	0.053556076
Coumestan	CA4	P22748	0.053556076
Coumestan	CA13	Q8N1Q1	0.053556076
Coumestan	CA5B	Q9Y2D0	0.053556076
Coumestan	CA5A	P35218	0.053556076
Coumestan	MAOA	P21397	0.053556076
Coumestan	ALOX5	P09917	0.053556076
Coumestan	CBR1	P16152	0.053556076
daidzein	ALDH2	P05091	1
daidzein	ESR1	P03372	1
daidzein	CA7	P43166	1
daidzein	ESR2	Q92731	1

daidzein	CA12	O43570	1
daidzein	CA4	P22748	1
daidzein	TBXAS1	P24557	0.52833091
daidzein	MAOA	P21397	0.52833091
daidzein	EGFR	P00533	0.52833091
daidzein	MGAM	O43451	0.52833091
daidzein	HTR2A	P28223	0.52833091
daidzein	HTR2C	P28335	0.52833091
daidzein	ADORA1	P30542	0.52833091
daidzein	ADORA2A	P29274	0.52833091
daidzein	HSD17B1	P14061	0.52833091
daidzein	ESRRA	P11474	0.52833091
daidzein	ESRRB	O95718	0.52833091
daidzein	ABCG2	Q9UNQ0	0.52833091
daidzein	CYP19A1	P11511	0.387023621
daidzein	ALOX12	P18054	0.300567522
daidzein	TYR	P14679	0.292767344
daidzein	MIF	P14174	0.292767344
daidzein	XDH	P47989	0.229907127
daidzein	PFKFB3	Q16875	0.206337197
daidzein	IL2	P60568	0.198443988
daidzein	CBR1	P16152	0.182723414
daidzein	HSD17B2	P37059	0.167041662
daidzein	ABCB1	P08183	0.151315357
daidzein	PTPRS	Q13332	0.143473412
daidzein	ALOX15	P16050	0.143473412
daidzein	PTGS1	P23219	0.127750341
daidzein	SLC6A2	P23975	0.127750341
daidzein	NOX4	Q9NPH5	0.127750341
daidzein	MAOB	P27338	0.119895127
daidzein	PON1	P27169	0.119895127
daidzein	CA2	P00918	0.112041901
daidzein	CA1	P00915	0.112041901
daidzein	STS	P08842	0.112041901
daidzein	ACHE	P22303	0.112041901
daidzein	TLR9	Q9NR96	0.112041901
daidzein	PPARA	Q07869	0.112041901
daidzein	ALOX5	P09917	0.112041901
daidzein	TNKS2	Q9H2K2	0.112041901
daidzein	TNKS	O95271	0.112041901
daidzein	PTPN1	P18031	0.112041901
daidzein	CA9	Q16790	0.112041901
daidzein	PLAT	P00750	0.112041901

daidzein	F10	P00742	0.112041901
daidzein	PLAU	P00749	0.112041901
daidzein	CA5B	Q9Y2D0	0.112041901
daidzein	PPARG	P37231	0.112041901
daidzein	MCL1	Q07820	0.112041901
dimethylesculetin	HSD17B3	P37058	0.33826033
dimethylesculetin	CA12	O43570	0.147464601
dimethylesculetin	CA9	Q16790	0.147464601
dimethylesculetin	ESR1	P03372	0.07148084
dimethylesculetin	ESR2	Q92731	0.07148084
dimethylesculetin	MIF	P14174	0.061946622
dimethylesculetin	AKR1B1	P15121	0.042894212
dimethylesculetin	ERN1	O75460	0.042894212
dimethylesculetin	EGFR	P00533	0.042894212
dimethylesculetin	QDPR	P09417	0.042894212
dimethylesculetin	CA2	P00918	0.042894212
dimethylesculetin	CA7	P43166	0.042894212
dimethylesculetin	CA1	P00915	0.042894212
dimethylesculetin	CA14	Q9ULX7	0.042894212
dimethylesculetin	ALDH2	P05091	0.042894212
dimethylesculetin	MAOB	P27338	0.042894212
dimethylesculetin	PON1	P27169	0.042894212
dimethylesculetin	SNCA	P37840	0.042894212
dimethylesculetin	COMT	P21964	0.042894212
dimethylesculetin	MB	P02144	0.042894212
dimethylesculetin	NQO1	P15559	0.042894212
dimethylesculetin	CA3	P07451	0.042894212
dimethylesculetin	RNASEH1	O60930	0.042894212
dimethylesculetin	XDH	P47989	0.042894212
dimethylesculetin	CXCR1	P25024	0.042894212
dimethylesculetin	DAO	P14920	0.042894212
dimethylesculetin	NAT1	P18440	0.042894212
dimethylesculetin	CCNE1	P24864	0.042894212
dimethylesculetin	KCNMA1	Q12791	0.042894212
dimethylesculetin	PTK2B	Q14289	0.042894212
dimethylesculetin	ALPL	P05186	0.042894212
dimethylesculetin	PLAA	Q9Y263	0.042894212
dimethylesculetin	GPR35	Q9HC97	0.042894212
dimethylesculetin	CCND1	P24385	0.042894212
dimethylesculetin	FLT4	P35916	0.042894212
dimethylesculetin	INSR	P06213	0.042894212
dimethylesculetin	CDK2	P24941	0.042894212
dimethylesculetin	PTK2	Q05397	0.042894212

dimethylesculetin	PLK1	P53350	0.042894212
dimethylesculetin	CSNK2A1	P68400	0.042894212
dimethylesculetin	TEK	Q02763	0.042894212
dimethylesculetin	BACE1	P56817	0.042894212
dimethylesculetin	EPHB4	P54760	0.042894212
dimethylesculetin	HSPA1A	P0DMV8	0.042894212
dimethylesculetin	NUAK1	O60285	0.042894212
dimethylesculetin	FGR	P09769	0.042894212
dimethylesculetin	LYN	P07948	0.042894212
dimethylesculetin	PNMT	P11086	0.042894212
dimethylesculetin	CLK1	P49759	0.042894212
dimethylesculetin	DYRK1B	Q9Y463	0.042894212
dimethylesculetin	AXL	P30530	0.042894212
dimethylesculetin	STS	P08842	0.042894212
dimethylesculetin	SRC	P12931	0.042894212
dimethylesculetin	IDO1	P14902	0.042894212
formononetin	IL2	P60568	1
formononetin	CYP19A1	P11511	0.288928252
formononetin	ALDH2	P05091	0.182484829
formononetin	ESR1	P03372	0.174270753
formononetin	CA12	O43570	0.174270753
formononetin	ADORA1	P30542	0.149732594
formononetin	ADORA2A	P29274	0.149732594
formononetin	TBXAS1	P24557	0.149732594
formononetin	MAOA	P21397	0.149732594
formononetin	MGAM	O43451	0.149732594
formononetin	HTR2A	P28223	0.149732594
formononetin	HTR2C	P28335	0.149732594
formononetin	ESRRA	P11474	0.149732594
formononetin	ESRRB	O95718	0.149732594
formononetin	ABCG2	Q9UNQ0	0.149732594
formononetin	CA7	P43166	0.141522086
formononetin	HSD17B2	P37059	0.141522086
formononetin	CA4	P22748	0.141522086
formononetin	CBR1	P16152	0.141522086
formononetin	SLC6A2	P23975	0.133337961
formononetin	ALOX12	P18054	0.133337961
formononetin	EGFR	P00533	0.125142649
formononetin	TYR	P14679	0.116965063
formononetin	XDH	P47989	0.116965063
formononetin	PFKFB3	Q16875	0.116965063
formononetin	ESR2	Q92731	0.108770969
formononetin	PTPRS	Q13332	0.108770969

formononetin	HSD17B1	P14061	0.108770969
formononetin	ABCB1	P08183	0.108770969
formononetin	ALOX15	P16050	0.108770969
formononetin	MIF	P14174	0.100578902
formononetin	PPARA	Q07869	0.100578902
formononetin	PTGS1	P23219	0.100578902
formononetin	TLR9	Q9NR96	0.100578902
formononetin	MAOB	P27338	0.100578902
formononetin	CA2	P00918	0.100578902
formononetin	CA1	P00915	0.100578902
formononetin	NOX4	Q9NPH5	0.100578902
formononetin	PON1	P27169	0.100578902
formononetin	TNKS2	Q9H2K2	0.100578902
formononetin	TNKS	O95271	0.100578902
formononetin	ACHE	P22303	0.100578902
formononetin	CA3	P07451	0.100578902
formononetin	CA6	P23280	0.100578902
formononetin	CA14	Q9ULX7	0.100578902
formononetin	CA13	Q8N1Q1	0.100578902
formononetin	CA5B	Q9Y2D0	0.100578902
formononetin	CA5A	P35218	0.100578902
formononetin	DHODH	Q02127	0.100578902
formononetin	STS	P08842	0.100578902
formononetin	ERCC5	P28715	0.100578902
formononetin	FEN1	P39748	0.100578902
formononetin	RAF1	P04049	0.100578902
formononetin	CA9	Q16790	0.100578902
formononetin	PTPN1	P18031	0.100578902
formononetin	BACE1	P56817	0.100578902
Scoparone	CA12	O43570	0.480888333
Scoparone	CA9	Q16790	0.480888333
Scoparone	CA13	Q8N1Q1	0.395234372
Scoparone	CA7	P43166	0.33826033
Scoparone	CA1	P00915	0.204771117
Scoparone	CA14	Q9ULX7	0.204771117
Scoparone	CA6	P23280	0.185814992
Scoparone	CA5B	Q9Y2D0	0.138112934
Scoparone	CA5A	P35218	0.109507901
Scoparone	CA4	P22748	0.090533089
Scoparone	XDH	P47989	0.061946622
Scoparone	CA2	P00918	0.05242396
Scoparone	EGFR	P00533	0.05242396
Scoparone	SRD5A1	P18405	0.05242396

Scoparone	ALOX5	P09917	0.042894212
Scoparone	CBR1	P16152	0.042894212
Scoparone	CA3	P07451	0.042894212
Scoparone	SRC	P12931	0.042894212
Scoparone	MAOA	P21397	0.042894212
Scoparone	AKR1C1	Q04828	0.042894212
Scoparone	MAOB	P27338	0.042894212
Scoparone	GSK3B	P49841	0.042894212
Scoparone	AKR1B1	P15121	0.042894212
Scoparone	PTGS2	P35354	0.042894212
Scoparone	KCNA3	P22001	0.042894212
Scoparone	IGF1R	P08069	0.042894212
Scoparone	KDR	P35968	0.042894212
Scoparone	AURKA	O14965	0.042894212
Scoparone	ERBB2	P04626	0.042894212
Scoparone	ESR2	Q92731	0.042894212
Scoparone	PDE5A	O76074	0.042894212
Scoparone	PDE3A	Q14432	0.042894212
Scoparone	PDE3B	Q13370	0.042894212
Scoparone	GPR35	Q9HC97	0.042894212
Scoparone	SQLE	Q14534	0.042894212
Scoparone	FGR	P09769	0.042894212
Scoparone	LYN	P07948	0.042894212
Scoparone	ADAMTS5	Q9UNA0	0.042894212
Scoparone	BACE1	P56817	0.042894212
Scoparone	PARP1	P09874	0.042894212
Scoparone	PARP2	Q9UGN5	0.042894212
Scoparone	MAPK8	P45983	0.042894212
Scoparone	MPO	P05164	0.042894212
Scoparone	AURKB	Q96GD4	0.042894212
Scoparone	CDK2	P24941	0.042894212
Scoparone	TYMP	P19971	0.042894212
Scoparone	GRM4	Q14833	0.042894212
Scoparone	CDC7	O00311	0.042894212
Scoparone	IKBKB	O14920	0.042894212
Scoparone	KCNN1	Q92952	0.042894212
Scoparone	KCNN3	Q9UGI6	0.042894212
Scoparone	KCNN2	Q9H2S1	0.042894212
Scoparone	CTSK	P43235	0.042894212
Scoparone	CTSS	P25774	0.042894212
Scoparone	CTSL	P07711	0.042894212
Scoparone	PDGFRB	P09619	0.042894212
Scoparone	FLT4	P35916	0.042894212

Scoparone	INSR	P06213	0.042894212
Scoparone	TEK	Q02763	0.042894212
Scoparone	EPHB4	P54760	0.042894212
Scoparone	CES1	P23141	0.042894212
Scoparone	MAPK10	P53779	0.042894212
Scoparone	CES2	O00748	0.042894212
Scoparone	EPHB3	P54753	0.042894212
Scoparone	PLK1	P53350	0.042894212
Scoparone	MAP2K3	P46734	0.042894212
Scoparone	BTK	Q06187	0.042894212
Scoparone	SYK	P43405	0.042894212
Scoparone	MAPK14	Q16539	0.042894212
Scoparone	ROCK2	O75116	0.042894212
Scoparone	CLK4	Q9HAZ1	0.042894212
Scoparone	RPS6KA5	O75582	0.042894212
Scoparone	RPS6KB1	P23443	0.042894212
Scoparone	PIM2	Q9P1W9	0.042894212
Scoparone	PBK	Q96KB5	0.042894212
Scoparone	PIM3	Q86V86	0.042894212
Scoparone	NTRK3	Q16288	0.042894212
Scoparone	PRKX	P51817	0.042894212
Scoparone	MAP4K4	O95819	0.042894212
Scoparone	BRAF	P15056	0.042894212
Scoparone	PTPRC	P08575	0.042894212
Scoparone	MAP3K14	Q99558	0.042894212
Scoparone	JAK2	O60674	0.042894212
Scoparone	TYK2	P29597	0.042894212
Scoparone	DYRK1B	Q9Y463	0.042894212
Scoparone	MET	P08581	0.042894212
Scoparone	ICAM1	P05362	0.042894212
Scoparone	SELE	P16581	0.042894212
Scoparone	NQO2	P16083	0.042894212
Scoparone	TGM2	P21980	0.042894212
Scoparone	PTPN13	Q12923	0.042894212
Scoparone	MCL1	Q07820	0.042894212
Scoparone	KDM4C	Q9H3R0	0.042894212
Scoparone	METAP1	P53582	0.042894212
Scoparone	APEX1	P27695	0.042894212
Scoparone	NUAK1	O60285	0.042894212
Scoparone	MAPKAPK2	P49137	0.042894212
Scoparone	MPI	P34949	0.042894212
Scoparone	JAK1	P23458	0.042894212
Scoparone	GRM5	P41594	0.042894212

Sitosterol	NPC1L1	Q9UHC9	0.894285595
Sitosterol	NR1H3	Q13133	0.705989664
Sitosterol	RORC	P51449	0.564356791
Sitosterol	HMGCR	P04035	0.514371374
Sitosterol	SHBG	P04278	0.45601846
Sitosterol	CYP51A1	Q16850	0.381040074
Sitosterol	CYP17A1	P05093	0.356071235
Sitosterol	CYP19A1	P11511	0.314405075
Sitosterol	SREBF2	Q12772	0.314405075
Sitosterol	AR	P10275	0.264448566
Sitosterol	RORA	P35398	0.181090099
Sitosterol	ESR1	P03372	0.16447208
Sitosterol	ESR2	Q92731	0.16447208
Sitosterol	PTPN1	P18031	0.139453236
Sitosterol	CYP2C19	P33261	0.131155749
Sitosterol	SLC6A2	P23975	0.12282732
Sitosterol	ACHE	P22303	0.11449479
Sitosterol	SERPINA6	P08185	0.11449479
Sitosterol	G6PD	P11413	0.11449479
Sitosterol	BCHE	P06276	0.11449479
Sitosterol	SLC6A4	P31645	0.106165761
Sitosterol	CHRM2	P08172	0.106165761
Sitosterol	NR1I3	Q14994	0.106165761
Sitosterol	NR1H2	P55055	0.106165761
Sitosterol	DHCR7	Q9UBM7	0.106165761
Sitosterol	PTGER1	P34995	0.106165761
Sitosterol	PTGER2	P43116	0.106165761
Sitosterol	VDR	P11473	0.106165761
Sitosterol	TBXAS1	P24557	0.106165761
Sitosterol	PTGES	O14684	0.106165761
Sitosterol	PPARD	Q03181	0.106165761
Sitosterol	CES2	O00748	0.106165761
Sitosterol	HSD11B1	P28845	0.106165761
Sitosterol	SQLE	Q14534	0.106165761
Sitosterol	PTPN6	P29350	0.106165761
Sitosterol	PTPN2	P17706	0.106165761
Sitosterol	GLRA1	P23415	0.106165761
Sitosterol	NOS2	P35228	0.106165761
Sitosterol	PPARG	P37231	0.106165761
Sitosterol	UGT2B7	P16662	0.106165761
Sitosterol	POLB	P06746	0.106165761
Puerarin	CA7	P43166	1
Puerarin	CA12	O43570	1

Puerarin	AKR1B1	P15121	0.11449479
Puerarin	SLC29A1	Q99808	0.106165761
Capronic acid	SLC22A6	Q4U2R8	0.248387034
Capronic acid	FABP4	P15090	0.187830848
Capronic acid	FABP3	P05413	0.187830848
Capronic acid	FABP5	Q01469	0.187830848
Capronic acid	PPARD	Q03181	0.187830848
Capronic acid	FFAR1	O14842	0.187830848
Capronic acid	FABP2	P12104	0.187830848
Capronic acid	HSD11B1	P28845	0.135105168
Capronic acid	AR	P10275	0.105843861
Capronic acid	VDR	P11473	0.105843861
Capronic acid	NR1H4	Q96RI1	0.105843861
Capronic acid	POLB	P06746	0.105843861
Capronic acid	CDC25A	P30304	0.105843861
Capronic acid	GPBAR1	Q8TDU6	0.105843861
Capronic acid	AKR1B10	O60218	0.105843861
Capronic acid	PPARA	Q07869	0.095255918
Capronic acid	PHF8	Q9UPP1	0.084973702
Capronic acid	KDM5C	P41229	0.084973702
Capronic acid	UGT2B7	P16662	0.084973702
Capronic acid	CYP19A1	P11511	0.074316474
Capronic acid	SERPINA6	P08185	0.074316474
Capronic acid	SHBG	P04278	0.074316474
Capronic acid	HSD17B3	P37058	0.074316474
Capronic acid	G6PD	P11413	0.074316474
Capronic acid	GABBR1	Q9UBS5	0.074316474
Capronic acid	PTGER2	P43116	0.064951087
Capronic acid	PTGFR	P43088	0.064951087
Capronic acid	FNTA	P49354	0.064951087
Capronic acid	NPC1L1	Q9UHC9	0.054487947
Capronic acid	GABRA2	P47869	0.054487947
Capronic acid	HSD11B2	P80365	0.044308319
Capronic acid	HAO1	Q9UJM8	0.044308319
Capronic acid	KDM2A	Q9Y2K7	0.03397069
Capronic acid	PLG	P00747	0.03397069
Capronic acid	PTPN1	P18031	0.03397069
Capronic acid	GSTK1	Q9Y2Q3	0.03397069
Capronic acid	LTA4H	P09960	0.03397069
Capronic acid	CDC45	O75419	0.023832743
Capronic acid	PTPRC	P08575	0.023832743
Capronic acid	CA2	P00918	0.023832743
Capronic acid	CA1	P00915	0.023832743

Capronic acid	HMGCR	P04035	0.023832743

Supplementary Table S4: 201 common gene targets between RP and CC.

Common targets	Common targets	Common targets
TYR	G6PD	PDE3B
MIF	GABBR1	SQLE
CYP19A1	PTGER2	ADAMTS5
CA7	PTGFR	PARP1
HSD17B2	NPC1L1	PARP2
CA12	HSD11B2	MAPK8
CA4	HAO1	MPO
CBR1	KDM2A	AURKB
ABCB1	PLG	TYMP
PPARA	GSTK1	GRM4
ESR1	LTA4H	IKBKB
ESR2	CDC45	KCNN1
EGFR	PTPRC	KCNN3
PTPRS	HMGCR	KCNN2
TLR9	NFKB1	CTSK
HSD17B1	CA3	CTSS
ALDH2	CA6	CTSL
MAOB	CA14	PDGFRB
ALOX12	CA9	CES1
TBXAS1	CA13	MAPK10
MAOA	CA5A	CES2
MGAM	ALOX5	EPHB3
HTR2A	TNKS2	MAP2K3
HTR2C	TNKS	BTK
ADORA1	PPARG	SYK
ADORA2A	AKR1B1	MAPK14
ESRRA	ERN1	ROCK2
ESRRB	QDPR	CLK4
ABCG2	SNCA	RPS6KA5
CA2	COMT	RPS6KB1
XDH	MB	PIM2
PTGS1	NQO1	PBK
SLC6A2	CXCR1	PIM3
PFKFB3	DAO	NTRK3
IL2	NAT1	PRKX
NOX4	KCNMA1	MAP4K4
ALOX15	PTK2B	BRAF
CA1	ALPL	MAP3K14
PON1	PLAA	JAK2

ACHE	GPR35	TYK2
PLAT	FLT4	MET
F10	INSR	ICAM1
PLAU	PTK2	SELE
CDC7	PLK1	NQO2
PTPN1	CSNK2A1	TGM2
ABCC1	TEK	PTPN13
CYP1B1	BACE1	KDM4C
STS	EPHB4	METAP1
HSP90AB1	HSPA1A	APEX1
DUSP3	NUAK1	MAPKAPK2
CDC25B	FGR	MPI
MCL1	LYN	JAK1
SLC22A6	PNMT	GRM5
FABP4	CLK1	NR1H3
FABP3	DYRK1B	RORC
FABP5	AXL	CYP51A1
PPARD	SRC	CYP17A1
FFAR1	IDO1	SREBF2
FABP2	DHODH	RORA
HSD11B1	ERCC5	CYP2C19
AR	FEN1	BCHE
VDR	RAF1	SLC6A4
NR1H4	SRD5A1	CHRM2
POLB	AKR1C1	NR1I3
CDC25A	GSK3B	NR1H2
GPBAR1	PTGS2	DHCR7
AKR1B10	KCNA3	PTGER1
PHF8	IGF1R	PTGES
KDM5C	KDR	PTPN6
UGT2B7	AURKA	PTPN2
SERPINA6	ERBB2	GLRA1
SHBG	PDE5A	NOS2
HSD17B3	PDE3A	SLC29A1

Supplementary Table S5: The first 20 KEGG pathways information.

Description	p.adjust	GeneSympol	Count
PI3K-Akt signaling pathway	-10.53	CHRM2,EGFR,ERBB2,FLT4,GSK3B,HSP90AB1,IGF1R,IKBKB,IL2,INSR,JAK1,JAK2,KDR,MCL1,MET,NFKB1,PDGFRB,PTK2,RAF1,RPS6KB1,SYK,TEK,ADORA2A,BRAF,MAPK14,MAP2K3,SRC,CSNK2A1,PTPN1,PTPN6,MAPK8,MAPK10,HSPA1A,CXCR1	22

MicroRNAs in cancer	-7.63	CDC25A,CDC25B,CYP1B1,EGFR,ERBB2,IKBKB,MCL1,MET,ABCC1,NFKB1,PDGFRB,ABCB1,PLAU,PTGS2,RAF1,RPS6KA5	16
EGFR tyrosine kinase inhibitor resistance	-12.72	AXL,BRAF,EGFR,ERBB2,GSK3B,IGF1R,JAK1,JAK2,KDR,MET,PDGFRB,RAF1,RPS6KB1,SRC,AR,HSP90AB1,IKBKB,NFKB1,NOS2,PPARD,PPARG,MAPK8,MAPK10,PTGER1,PTGER2,PTGS2,PTK2,ROCK2,MAPK14,ESR1,ESR2,CTSL,PLAU,PTPN6,FLT4,CDC25A,CDC25B,PDE3B,PLK1,TYMP,RPS6KA5,INSR,PTPN1,G6PD,NTRK3,GABBR1,HSPA1A,GRM5,HTR2A,HTR2C,ERN1,CHRM2,LYN	14
Serotonergic synapse	-10.42	ALOX12,ALOX5,ALOX15,BRAF,CYP2C19,HTR2A,HTR2C,KCNN2,MAOA,MAOB,PTGS1,PTGS2,RAF1,SLC6A4,CBR1,LTA4H,TBXAS1,PTGES	14
Apoptosis	-9.63	PARP1,CTSK,CTSL,CTSS,ERN1,IKBKB,MCL1,NFKB1,MAPK8,MAPK10,PTPN13,RAF1,MAP3K14,PARP2,MAPK14,EGFR,CXCR1,LYN,MET,SRC,CSNK2A1,FGR,GSK3B,HSPA1A,ICAM1,JAK1,MAP2K3,SYK,TYK2,NQO1,HSP90AB1,KDR,PLAT,PTK2,SELE,CYP17A1,ESR1,ESR2,JAK2,BRAF,CDC25B,DUSP3,PDGFRB,RPS6KA5,MAPKAPK2,MAP4K4,PPARA,NR1H3,IL2,RORA,RORC,ALOX5,NOS2,PLG,BTK,PPARG,PTGS2,INSR,PTPN1,RPS6KB1,NR1H2,PTK2B,VDR,TLR9,ERBB2,NTRK3,PTGS1,ALOX12,HTR2A,HTR2C,PTGER2,ROCK2,NOX4,ADORA1,ABCC1,POLB,GRM5	14
cAMP signaling pathway	-8.05	ADORA1,ADORA2A,BRAF,CHRM2,GABBR1,NFKB1,PDE3A,PDE3B,PPARA,MAPK8,MAPK10,PTGER2,RAF1,ROCK2,FABP4,INSR,PTGS1,PTGS2,KCNMA1,PDE5A	14
Steroid hormone biosynthesis	-12.94	STS,COMT,CYP1B1,CYP17A1,CYP19A1,AKR1C1,HSD11B1,HSD11B2,HSD17B1,HSD17B3,HSD17B2,SRD5A1,UGT2B7,ALOX5,IGF1R,INSR,PTGS2	13
Chemokine signaling pathway	-7.6	BRAF,PTK2B,FGR,GSK3B,IKBKB,CXCR1,JAK2,LYN,NFKB1,PTK2,RAF1,SRC,ROCK2,MAPK14,KDR,PTGS2,MAPKAPK2,EPHB3,EPHB4,MET	13
drug metabolism	-8.57	NAT1,CES1,CYP2C19,TYMP,MAOA,MAOB,MPO,SLC6A2,UGT2B7,XDH,CES2,GSTK1	12
Calcium signaling pathway	-6.67	ADORA2A,CHRM2,EGFR,ERBB2,PTK2B,GRM5,HTR2A,HTR2C,NOS2,PDGFRB,PTGER1,PTGFR,ADORA1,GABBR1,GLRA1,GPR35,GRM4,PLG,PTGER2	12
Nitrogen metabolism	-16.66	CA1,CA2,CA3,CA4,CA5A,CA6,CA7,CA9,CA12,CA14,CA13	11

NF-kappa B signaling pathway	-8.4	PARP1,BTK,CSNK2A1,ICAM1,IKBKB,LYN,NFKB1,PLAU,PTGS2,SYK,MAP3K14	11
Phospholipase D signaling pathway	-5.75	EGFR,PTK2B,GRM4,GRM5,CXCR1,INSR,PDGFRB,PTGFR,RAF1,SYK,BRAF,ICAM1,PTPN6	10
Chemical carcinogenesis	-6.5	NAT1,CBR1,CYP1B1,CYP2C19,HSD11B1,PTGS2,SLC6A2,UGT2B7,GSTK1,AKR1C1	9
HIF-1 signaling pathway	-5.53	EGFR,ERBB2,IGF1R,INSR,NFKB1,NOS2,PFKFB3,RPS6KB1,TEK,HSPA1A,PPARG,HMGCR	9
B cell receptor signaling pathway	-6.13	BTK,GSK3B,IKBKB,LYN,NFKB1,PTPN6,RAF1,SYK,MAPK14,IL2,PTPRC,MAP3K14,BRAF,PPARD,RPS6KB1,PIM2	8
PPAR signaling pathway	-6.09	FABP4,FABP2,FABP3,FABP5,PPARA,PPARD,PPARG,NR1H3	8
Leishmania infection	-4.98	MAPK14,JAK1,JAK2,NFKB1,NOS2,PTGS2,PTPN6,IKBKB,PTK2,AKR1C1	7
Tyrosine metabolism	-5.68	COMT,MAOA,MAOB,MIF,PNMT,TYR,ALDH2,CYP1B1,IDO1,DAO,NOS2,MAPK14,GSK3B,MAPK8,MAPK10,CYP2C19,UGT2B7,GSTK1,NFKB1	6
Antifolate resistance	-4.62	ALOX12,IKBKB,ABCC1,NFKB1,ABCG2,ABCB1	5
